# Unmet Need for Emergency Medical Services in Hanoi, Vietnam

**DOI:** 10.31662/jmaj.2020-0110

**Published:** 2021-07-06

**Authors:** Bui Hai Hoang, Thi Hue Mai, Thai Son Dinh, Thanh Nguyen, Trung Anh Dang, Van Cuong Le, Quoc Chinh Luong, Shinji Nakahara

**Affiliations:** 1 Emergency and Critical Care Department, Hanoi Medical University Hospital, Hanoi, Vietnam; 2 Emergency and Critical Care Department, Hanoi Medical University, Hanoi, Vietnam; 3 Thanh Hoa Campus, Hanoi Medical University, Thanh Hoa, Vietnam; 4 Institute for Preventive Medicine and Public Health, Hanoi Medical University, Hanoi, Vietnam; 5 115 Center of Hanoi, Hanoi, Vietnam; 6 Cardiovascular Intervention Center, Thanh Hoa General Hospital, Thanh Hoa, Vietnam; 7 Center For Emergency Medicine, Bach Mai Hospital, Hanoi, Vietnam; 8 Graduate School of Health Innovation, Kanagawa University of Human Services, Kawasaki, Japan

**Keywords:** emergency medical services, low- and middle-income countries, access to healthcare, strengthening health system, Vietnam

## Abstract

Low- and middle-income countries urgently need to improve emergency medical services (EMSs) as a component of their healthcare systems. Here, we detailed EMS resources and their provision in Hanoi, Vietnam, and discussed necessary policies to upgrade EMSs. Between 2013 and 2018, EMS resources, measured as provider-to-population and ambulance-to-population ratios, decreased, whereas service provision, measured as the number of patients transported by ambulance per population, increased. EMS resources and their provision in Hanoi are far below the standards of high-income countries or figures in neighboring Asian countries. Therefore, it is imperative to upgrade health policies for the appropriate allocation of healthcare resources to EMSs and hospital services.

## Introduction

Emergency medical services (EMSs) are the first points of contact with health services for people with acute life-threatening conditions as they provide first aid and transport patients to medical facilities. In many low- and middle-income countries (LMICs), the number of patients with various life-threatening conditions is increasing because of the acute onset of noncommunicable diseases and injuries. Despite this, patients frequently face problems such as remote hospitals, insufficient EMS functions, and lack of transportation ^(1)^.

Vietnam has a growing demand for EMSs because of a rapid epidemiological transition in the 1990s: a decrease in the incidence of communicable diseases that used to be dominant causes of mortality (accounting for 52% of deaths in 1986 and 12% in 2018), an increase in the incidence of noncommunicable diseases (from 42% to 63%), and external causes, such as injuries (6% to 24%) during the same period ^(2)^. These changes have resulted in an increase in the number of patients who require emergency care due to conditions such as ischemic heart disease, stroke, and injuries. This is evident in urban areas such as Hanoi, the capital city of Vietnam, which experienced a rapid increase in population, residential areas, and road traffic (the population was 8.05 million in 2019). Nonetheless, Hanoi’s EMSs are unable to meet the increasing need for their services due to their lack of functionality, and most critical patients, including those with out-of-hospital cardiac arrest, have to travel to hospitals by themselves ^(3)^.

Healthcare communities and policy makers in LMICs urgently need to attend to the issue of unmet demands for EMSs. Thus, on the basis of the data from Hanoi, Vietnam, we detailed insufficient resources and current service provision in EMSs and discussed the type of resources that need to be allocated in such regions.

## EMS System in Hanoi and Its Resources and Service Provision

The city of Hanoi controls its EMS. It was established in 1975, following the French model of ambulances staffed with physicians and nurses, and it provides free services to the entire city. The call command center is named the “115 Hanoi Emergency Center,” after the universal three-digit phone number (115) for EMS calls nationwide. It dispatches an ambulance from the nearest of the five dispatch stations in Hanoi. The center and dispatch stations are all independent of hospitals, and the EMS physicians and nurses only provide care in the ambulances and do not work for hospitals.

From 2013 to 2018, the number of EMS resources in Hanoi has decreased according to data from the 115 Hanoi Emergency Centre (Table 1). The number of ambulances decreased (from 23 to 19), as did the number of physicians (from 52 to 43) and nurses (from 68 to 65). By contrast, the population of Hanoi increased by approximately 10% from 7.2 million to 7.8 million during this period. The provider-to-population ratio decreased from 1.7 to 1.4 per 100,000 people, and the ambulance-to-population ratio decreased from 0.32 to 0.24 per 100,000 people. Meanwhile, the need for service provision in the EMS increased. The number of patients transported by ambulances increased by approximately 45% (from 18,408 to 26,808). Similarly, the number of transported patients per population increased from 257 to 342 per 100,000 people. In 2018, the average response time between making the call and the arrival of the ambulance at the patient’s location was 14.5 min (the time data were only available for 2018).

**Table 1.** Resource and Service Provision in the EMS System and National Hospitals in Hanoi, Vietnam, from 2013 to 2018.

	2013	2014	2015	2016	2017	2018
Population	7,164,200	7,265,600	7,390,900	7,522,600	7,739,400	7,832,200
EMS resources						
Number of ambulances	23	23	22	22	19	19
Number of EMS physicians	52	49	44	52	48	43
Number of EMS nurses	68	67	66	64	61	65
Number of patients transported by EMS	18,408	20,373	20,825	22,110	26,004	26,808
Ambulances/100,000 population	0.32	0.32	0.30	0.29	0.25	0.24
Transported patients/100,000 population	256.9	280.4	281.8	293.9	336.0	342.3
Providers*/100,000 population	1.67	1.60	1.49	1.54	1.41	1.38

EMS, emergency medical services *Providers include physicians and nurses

## Discussion

The EMS resources in Hanoi do not meet the population’s needs. The ambulance-to-population ratio (0.24 per 100,000 people) was much lower than the standards for urban areas in high-income countries (HICs), i.e., 2-3.3 per 100,000 people ^(1)^ or ratios in some HICs at 0.8-3.2 per 100,000 people^(4)^, even lower than that of neighboring LMICs in the early 2010s (0.3 in Bangkok, Thailand, and 0.6 in Kuala Lumpur, Malaysia) ^(4)^. Similarly, the provider-to-population ratio was lower than that of HICs at 4-56 per 100,000 people ^(4)^. These shortcomings worsened over recent years because the number of resources decreased while the population increased, possibly owing to insufficient budget allocation for vehicle maintenance and procurement and for staff recruitment and salary, despite the presence of adequate resources in tertiary care hospitals.

Moreover, the recruitment of new EMS personnel is facing several challenges. First, although physicians and nurses should undergo an 18-month clinical training program in inpatient settings after graduation to acquire their complete clinical license, EMSs are not considered inpatient facilities, which makes obtaining post-graduate training difficult. Second, their working conditions include environments such as highway accidents and violent scenes, exposing them to the risk of trauma.

The EMS response time of approximately 15 min in Hanoi is much longer than that of HICs (5-10 min) ^(4)^ or the standard response times in HICs (within 8 min for 90% of calls or 5 min for cardiac arrest cases) ^(5)^, if not extremely long compared to neighboring LMICs (11.8 min in Bangkok, Thailand; 22.5 min in Kuala Lumpur, Malaysia) ^(6)^. The delay in response primarily results from traffic congestion and drivers not making way for ambulances. Owing to this delay, people would rather use a taxi or private vehicle than wait for the EMS even when transporting critical patients, which would affect patient outcomes. A recent multicenter study in Hanoi showed that only 21% of cardiac arrest patients transported to tertiary care hospitals used EMSs ^(3)^.

Although service provision in EMSs is rapidly improving, the number of transported patients (342 per 100,000 people) is lower than that in HICs (2000-6000 per 100,000 people) or neighboring LMICs (e.g., 550 in Bangkok and 2900 in Kuala Lumpur) ^(4), (7)^. Despite regional variations in population structure and epidemiological situations, we can assume that similar needs for EMSs exist in Hanoi. Thus, the small number of transported patients implies that most of these needs remain unmet. Many LMICs share the same issue of low utilization of EMS.

Cooperation and communication between EMSs and emergency departments of tertiary hospitals are currently not sufficient. As a rule, the EMS personnel are supposed to select the nearest hospital suitable for the patients’ conditions and contact the emergency medical team of the selected hospital in advance so that they can prepare for the treatments. In reality, however, most transfers by EMS in Hanoi are made without prior notice.

Other areas of Vietnam have similar or even worse EMS situations, except Ho Chi Minh City, the largest city in the country, which has a control center and 34 dispatch stations. Among 63 provinces and large cities, only 15 have EMSs (115 emergency centers) with limited functions. Additionally, most of the 115 centers have outsourced their dispatch services to private entities, which means that untrained personnel are managing ambulance dispatch, such as taxi allocation.

People are reluctant or unable to use EMSs because of various barriers. An insufficient number of EMS units result in a longer response time that is aggravated by traffic congestion. Many people do not know how to use EMSs and lack awareness on the role of EMSs. Even if they are aware, people may not use EMSs because of their low quality of care. These barriers are particularly severe in remote areas. For example, patients must pay the cost of long-distance travel even when using an EMS, and reaching the hospital can take hours or even days. These barriers widen the gap between those who do and do not have good access to healthcare services.

In contrast to the scarce resources for EMS, hospital resources greatly increased in Hanoi during this period. The Ministry of Health and the City Government of Hanoi implemented a plan to upgrade and expand the existing 20 tertiary care hospitals and build 15 new hospitals with a total of 5000 beds by the year 2020. This plan includes the securing and training of many medical personnel to perform hospital services, which would cause an outflow of human resources from EMS.

Policymakers need to recognize the importance of EMSs as a component of the healthcare system and provide balanced resources to in-hospital and pre-hospital care components. EMSs contribute to improving access to emergency care as the first point of contact to healthcare by providing rapid transport to the hospital and providing first aid to patients at the scene. Quality hospital care can only be provided appropriately when people have good and equitable access to the care.

More resources should be allocated from hospital improvement projects to EMS development. For example, maintenance and procurement of equipment, facilities, and vehicles require a significant budget. To strengthen human resources, the disadvantages of EMS personnel can be minimized as follows: modifying the licensing system, improving staff training and supervision, increasing their salaries, and enhancing safety. When resources are insufficient to develop EMSs based on expensive HIC models, such as the French physician-based system, low-cost and basic, yet effective models should be adopted to increase EMS coverage. Paramedic-, medical assistant-, or volunteer-based systems implemented in other LMICs such as Thailand and Malaysia may also be useful in Vietnam ^(6)^. Additionally, improving public relations to raise people’s awareness of the roles of EMSs is crucial in garnering their support for these policies, appropriate EMS use, and facilitating EMS activities.

In conclusion, insufficient resources have resulted in large, unmet EMS needs in Hanoi. Policymakers should recognize the importance of EMS as a healthcare component and allocate additional balanced resources. They need to address the challenges faced by EMS personnel to ensure that EMS resources and provision indicators in Hanoi meet the standards of those in HICs.

## Article Information

### Conflicts of Interest

None

### Sources of Funding

This work was supported by the Japan Society for the Promotion of Science KAKENHI (16K11422 and 19K09403); the funding body did not play any role in conducting the study or preparing the manuscript.

### Author Contributions

BHH and SN conceived the idea; BHH, THM, TSD, TN, and TAD collected information, analyzed the data, and drafted the manuscript; and BHH, VCL, TN, QCL, and SN critically revised and rewrote the manuscript.

### Availability of Data

The data used in this study are public domain data available from the Hanoi dispatch center.

### Ethical Considerations/Informed Consent/Human Rights

Because this study used public domain aggregated data, requirements for ethical clearance and informed consent were waived.
